# Problem-Based Learning in North American Primary Care Postgraduate Medical Education: A Rapid Review

**DOI:** 10.7759/cureus.72818

**Published:** 2024-11-01

**Authors:** Megan Clemens, Susan Avery, Russell Dawe

**Affiliations:** 1 Faculty of Medicine, Memorial University of Newfoundland, St. John's, CAN

**Keywords:** case-based learning methods, family medicine residency, medical education, postgraduate medical education (pgme), primary care, problem-based learning (pbl)

## Abstract

Problem-based learning (PBL) in medical education is centered around a problem or case and is learner-led, involving small groups and problem-solving. PBL is ubiquitous in North American undergraduate medical education (UGME) due to reported learner satisfaction, efficacy, and long-term knowledge retention; however, its application to postgraduate medical education (PGME) is less defined. This review addresses the knowledge gap on the use and efficacy of PBL in PGME, specifically among primary care specialties due to their unique training needs, using the Kirkpatrick model as the theoretical basis for interpreting results. A search for articles using PubMed resulted in 17 selected articles that included primary care PGME learners undergoing at least one PBL session led by another learner. Learners were overwhelmingly satisfied with PBL, reporting increased confidence and comfort in the subject area. While none of the studies measured behavior change objectively, over half reported increased comfort in diagnosing, prescribing, and managing patients. This review extends the positive feedback found from PBL in UGME settings to apply to PGME and highlights the suitability of PBL for primary care due to increased confidence, learner satisfaction, perceived knowledge gain, and objective learning outcomes.

## Introduction and background

Problem-based learning (PBL) was first introduced by Dr. Howard Barrows in the 1970s for McMaster University’s undergraduate medical education (UGME) program [1]. Key elements of PBL include small groups, student-led learning, and a focus on problem-solving [2]. PBL is theoretically sound for training physicians as it simulates real-life scenarios [3]. Additionally, learning in groups is supported by the social learning concepts of modeling, where learners observe and imitate others’ actions, and scaffolding, a process where new knowledge is built step by step on prior understanding [4]. The development of PBL represents a shift from content-focused to process-focused learning, a significant advancement in medical education [2].

By the turn of the 21st century, most North American medical schools had adopted PBL [2], resulting in increased test scores compared to traditional teaching methods [5-7]. While students who learn via PBL are found to have less immediate knowledge, they are better able to apply their knowledge and show greater long-term retention [8,9]. Students also report being satisfied with PBL [10]. However, students scoring above the median benefit equally from both didactic lectures and PBL [6], indicating that PBL might not be a one-size-fits-all approach.

A gap in the literature exists regarding PBL’s impact on postgraduate medical education (PGME), referred to as graduate medical education (GME) in the United States, as most studies are focused on UGME [6]. PGME includes the training period after medical school, entailing internship, residency, and fellowship, lasting between two and eight years [11]. Previous studies have shown that traditional didactic lectures have mixed efficacy in PGME [12,13]. One reason that large-group learning has fallen out of favor is that residents are often located at dispersed learning sites and, even when they are all together, often face patient-care-related interruptions [14,15].

Theoretically, PBL is an excellent fit for PGME programs to replace or supplement the existing didactic curriculum. Unlike medical students, residents are less motivated by grades and more by patient care [16,17]. The small group environment may offer scheduling flexibility, accommodating busy PGME schedules [18]. These advantages are supported by an increased prevalence of the flipped classroom approach, which has a PBL component [19]. However, PBL in PGME still requires proper investigation, especially given residents’ clinical responsibilities, which limit out-of-class study hours [16].

One specific setting in which the understanding of PBL would be useful is in primary care specialties. Primary care has specific training needs due to its broad scope of practice compared to other, more specialized disciplines [20]. Additionally, they may have a shorter GME training period. For example, family medicine residency training lasts two or three years compared to five years or longer for surgical or medical subspecialty training [21]. The feasibility of PBL across different PGME specialties, particularly in primary care, remains uncertain [17].

This rapid review addresses the knowledge gap on the current use and efficacy of PBL in PGME programs to support timely decision-making in the upcoming academic year. This review emphasizes learner-led interventions as a core tenet of PBL, and primary care specialties are highlighted due to their unique training needs.

Definitions

Several terms relate to and are often used interchangeably with PBL, which falls under the umbrella of active learning. Case-based learning (CBL) is considered a subtype of PBL for this review as it is often described similarly in the literature but with a more specific application to medical settings. Other active learning types frequently involve PBL or CBL, such as flipped classrooms and team-based learning.

Theoretical model

**Figure 1:**
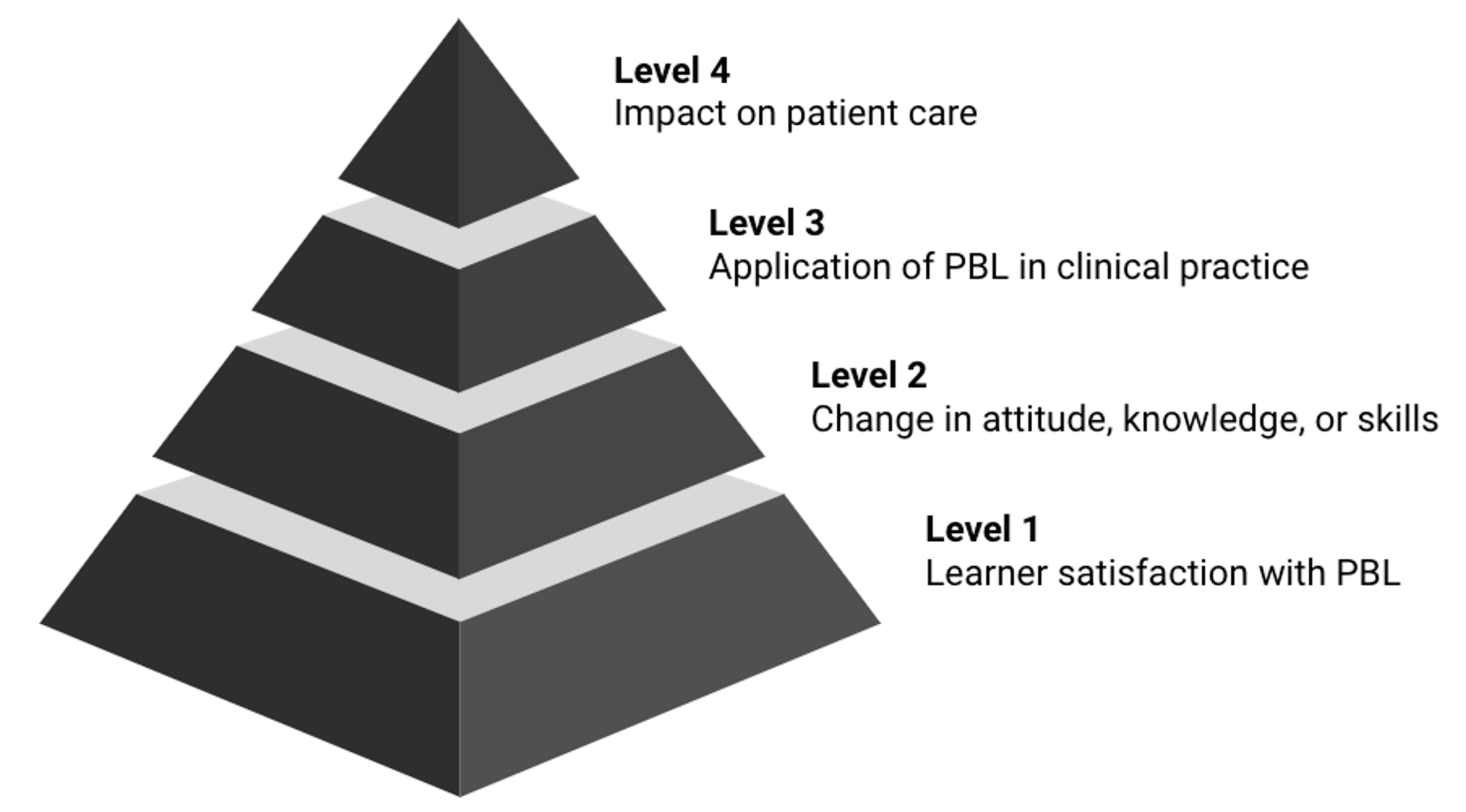
The Kirkpatrick model adapted for evaluating medical education PBL: Problem-based learning

Training program outcomes are categorized using the Kirkpatrick model. The original model [22] has since been adapted for evaluating medical education programs and specifically PBL for this context (Figure 1). This modified model includes learner satisfaction (Level 1), change in attitude and modification to knowledge or skills (Level 2), application of PBL in clinical practice (Level 3), and impact on patient care (Level 4) [23]. The Kirkpatrick model permits a structured approach to comprehensively evaluating outcomes of a medical training program, focusing not just on learner satisfaction but downstream impacts on the organization as a whole.

## Review

Methods

Search Strategy

A search for articles was conducted using the PubMed database on July 4, 2024. PubMed was selected as the sole database for this rapid review to balance the need for efficiency and timely publication with maintaining high research quality. The search strategy (Appendix 1) was developed with the support of a university librarian. Both Medical Subject Heading (MeSH) headings and free text were used. Two main concepts were searched to identify articles of interest: ‘postgraduate medical education (PGME)’ and ‘problem-based learning (PBL)’. PGME included the training of medical interns, residents, or fellows. PBL broadly included any training whose learning objectives were obtained through solving a problem or case.

Study Selection

The articles returned from the PubMed search were uploaded into Covidence software. Title and abstract screening was conducted by a single screener (MC) to ensure that articles met the searched terms of PGME and PBL, as well as the additional concepts, including primary care specialties and interventions that were learner-led and composed of small groups (Table 1). The included articles were restricted to those involving training programs in North America (i.e., Canada and the United States) for ease of comparison due to the heterogeneity of postgraduate medical training in other regions [24]. Only articles published in English were included. There were no restrictions to the publication date. The full-text screening was also conducted by the single screener (MC).

**Table 1:** Selection criteria for the review

	Inclusion	Exclusion
Date	Any	N/A
Country	Canada USA	Other countries
Participants	PGME Primary care discipline (e.g., family medicine, internal medicine, general pediatrics)	UGME Non-primary care disciplines Continuing professional development Allied health
Training structure	PBL or CBL Small groups (3-15 learners) Learner-led	Lecture-based Experiential or practice-based learning Large groups Instructor- or faculty led
Training delivery	Synchronous, in-person, virtual, or hybrid	Asynchronous
Outcome	Any (e.g., learner or faculty satisfaction, learner engagement, exam results)	N/A

Data Extraction

Data extraction was conducted using Excel by a single extractor (MC). Information about the population, intervention, control group (if applicable), and outcome were recorded. Data were reviewed by other authors (SA and RD) and refined by consensus after further discussion.

Quality Assessment

The quality of the articles was assessed using the Mixed Methods Appraisal Tool (MMAT) [25]. Each article was individually assessed for methodological quality using the MMAT template for its respective study type.

Findings

Summary of the Included Studies 

**Figure 2:**
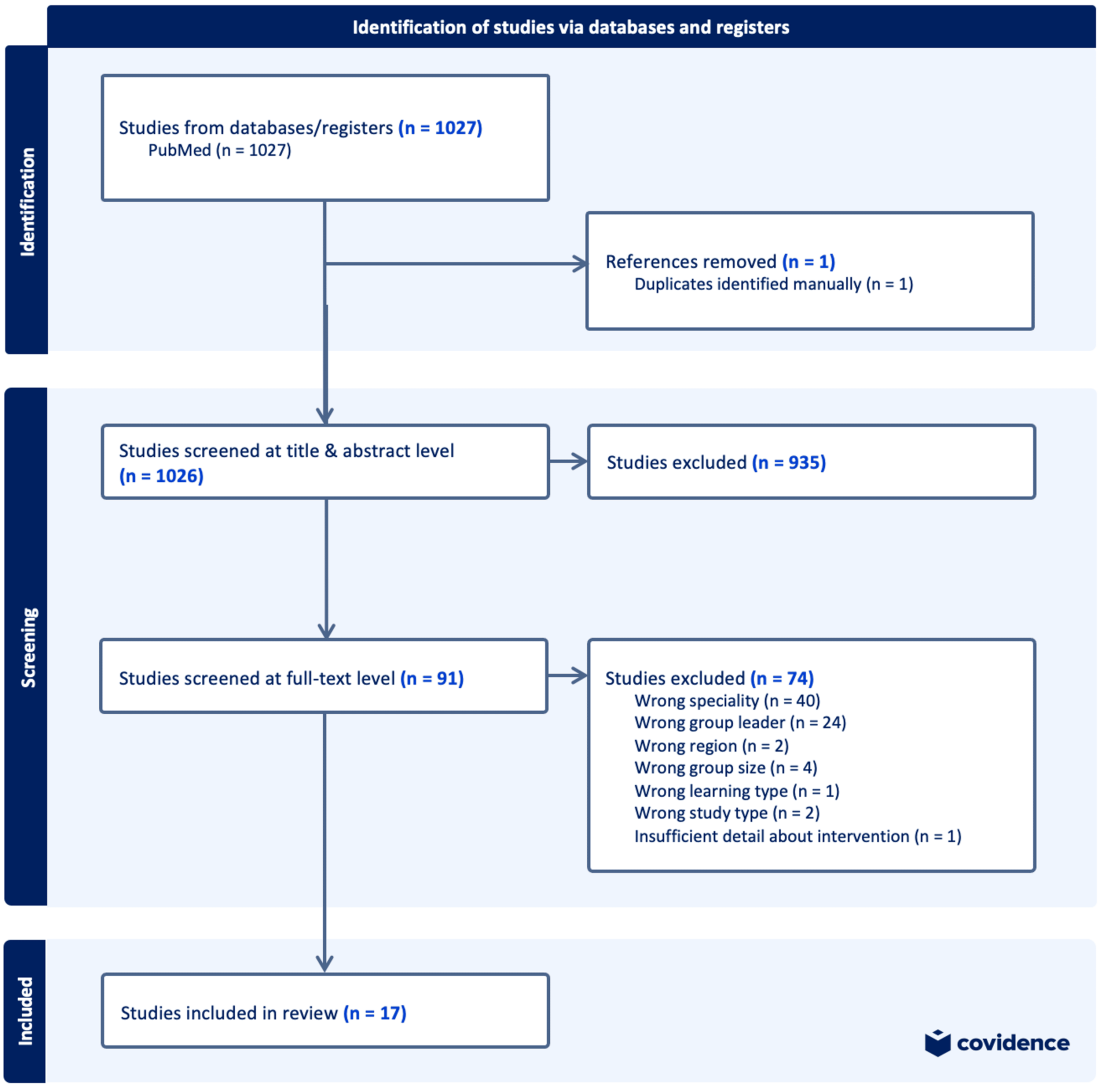
PRISMA flow diagram of the article selection process PRISMA: Preferred Reporting Items for Systematic Reviews and Meta-Analyses

There were 1,027 articles returned in the literature search that were compared to the criteria of the literature review (Figure 2). Of the 17 articles that met the criteria (Table 2), 16 were from institutions in the United States and one from Canada. Six articles focused on internal medicine, six on pediatrics, two on family medicine, and the remaining articles covered either all residents from the institution or those rotating through a particular service. Most interventions included all program years, though some targeted specific training levels, primarily the first postgraduate year of training (PGY-1). One program targeted PGY-1 and PGY-2, another only PGY-2, and one exclusively senior residents.

**Table 2:** Characteristics and findings of the included studies CBL: Case-based learning; PBL: Problem-based learning; PGY: Post-graduate year

Study	Country	Specialty	Intervention	# of sessions	Control	Evaluation	Outcomes	Quality
Agrawal et al., 2004 [26]	Canada	Family medicine	Debate and group discussions about pharmaceutical marketing	2	-	Pre- and post-intervention surveys	• Change in opinion about topic • No change in self-reported behavior or confidence	High
Ardoin et al., 2022 [27]	USA	Internal medicine	Small group discussions about internal medicine cases	4	-	Post-intervention survey	• Satisfied with content • Sessions perceived as effective, meeting learning objectives • Reported post-session independent study	High
Benson et al., 2018 [28]	USA	Various	PBL case study session about diabetes self-management, tailored to local context	1	Series of five didactic sessions	Pre- and post-didactic session surveys, post-PBL survey	• PBL perceived as mostly effective, high self-reported knowledge • Didactics rated good or average with no change in opinion on the topic	Moderate
Blair et al., 2020 [29]	USA	Internal medicine	Flipped classroom about pharmacotherapy for type 2 diabetes	1	-	Knowledge assessment at 3 time points, pre- and post-intervention survey, focus group	• High satisfaction • Incr. comfort and self-reported knowledge 6 months after intervention	High
Caton et al., 2019 [30]	USA	Internal medicine	Case-based modules to introduce PGY-1s to residency	8	-	Post-intervention survey	• Very high satisfaction • Incr. self-reported knowledge	High
Dickerman et al., 2018 [31]	USA	Various	Didactic session, reflection, and case scenarios about leadership in medicine	1	-	Post-intervention survey	• Moderate agreement that learning objectives were met	High
Dittus et al., 2014 [32]	USA	Various	Team-based activity and small group PBL sessions about common medical complaints	42	-	Pre- and post-intervention survey	• Incr. self-reported efficacy for most topics	High
El-Hallal et al., 2023 [33]	USA	Pediatrics	Didactic presentation, small groups, role play, and simulation about pediatric seizures	6	-	Pre- and post-intervention surveys	• Incr. objectively measured knowledge in attendees; greatest increase in PGY-1s • Incr. comfort with some diagnoses, prescribing	High
Harris et al., 2020 [34]	USA	Internal medicine	Daily session for one week about diagnostic steps, etc. followed by case presentations during morning report	Not specified	Cohorts prior to implementation	Pre- and post-intervention surveys	• Incr. objectively measured diagnostic reasoning (effect noted in PGY-1s and PGY-2s, but was greatest in PGY-1s)	High
Manning et al., 2023 [35]	USA	Pediatrics	Seminar followed by monthly case-based discussions about behavioral and mental health assessment	Not specified	-	Pre- and post-intervention surveys	• High satisfaction • Incr. self-reported knowledge	High
Ramadurai et al., 2021 [36]	USA	Internal medicine	Case discussion about medical needs and related social determinants of health immediately before rounds	Not specified	-	Pre- and post-intervention surveys, direct observation from social worker	• High comfort at baseline • Incr. comfort and objectively measured knowledge	Moderate
Sass et al., 2001 [37]	USA	Family medicine	Group project researching the region’s demographic information and health needs	Not specified	-	Discussion with uninvolved faculty member	• Incr. confidence using data • Incr. appreciation for determinants of health	Moderate
Sathe et al., 2022 [38]	USA	Pediatrics	PBL exercise at primary care clinic about transition of adolescents to adult healthcare	Not specified	-	Pre- and post-intervention survey	• Very high satisfaction • Incr. comfort in providing care transitions	High
Schulmeister et al., 2023 [39]	USA	Pediatrics	Didactic session, small group CBL, and peer teaching about insulin management	1	-	Pre- and post-intervention surveys	• Very high satisfaction • Incr. self-efficacy • Incr. objectively measured knowledge	High
Vertress et al., 2013 [40]	USA	Internal medicine	Didactic session and open-ended discussion about medical ethics cases	Not specified	-	Post-intervention survey	• Very high satisfaction and perceived efficacy • Especially valued debriefing	Moderate
Volerman et al., 2019 [41]	USA	Pediatrics	Clinical case exercise and large group discussion about specialty topics	3	-	Pre- and post-intervention surveys, knowledge assessment during intervention	• Satisfied • Reported more engagement than traditional teaching • Low pre-class reading completion	High
Warman et al., 2023 [42]	USA	Pediatrics	Case-based discussions about updated asthma treatment guidelines	1	-	Post-intervention survey	• Content perceived as relevant • Satisfied with case-based approach	High

Quality Assessment

Quality assessment using the MMAT revealed that the included studies were of high or moderate quality. Each study was evaluated according to its design (qualitative, quantitative, or mixed methods). While the sample sizes were often small (e.g., <50 participants), many studies their entire target population, for example, an intern cohort, minimizing the opportunity for selection bias. One common limitation was that the question design did not clearly address the study purpose. Additionally, most studies were at a single training site, limiting generalizability to other settings.

Training Program Logistics 

Programs ranged from single sessions to series over a year or longer. Each session was delivered in person, except for one hybrid program [33] and one that was either in-person, virtual, or hybrid, depending on the current COVID-19 restrictions [35]. Four programs were single sessions, lasting between 45 and 90 minutes [29,31,39,42]. Other programs had two [26], three [41], or four sessions [27]. Some programs adopted a “bolus/booster” pattern, involving an intense initial training period, followed by less frequent sessions. For example, one intervention included five sessions over one week with fewer regular sessions in the following months [34] and another featured a three-hour seminar followed by monthly 45-minute sessions on the same topic [35]. The longest program included 42 nearly weekly sessions over eleven months [30]. One longer intervention on medical ethics noted a subjective increase in learners’ confidence in discussing ethics and their use of appropriate terminology throughout the program [40]. This finding suggests the value of interventions spaced over time with recurring themes, permitting longitudinal learning.

Programs often occurred at times convenient to schedule; however, a few PBL programs occurred during specific “rotations” or training periods. For example, Sass et al. used PBL during the learners’ one-month community medicine rotation [37]. Their teaching session involved a small group project where learners worked together to analyze the health disparities in the community, allowing them to witness the relevance of their practice. Similarly, Ramadurai et al. described using PBL during a rotation in the intensive care unit [36]. Each session took place just before rounds, permitting learners to immediately and directly apply their knowledge. While not a specific rotation, Caton et al. described a program that occurred during the first three months of the first-year internal medicine residents’ training, serving as an introduction to residency [30].

Training Program Content

Most articles focused on medical topics, except one on leadership in medicine [31]. Single sessions addressed specific topics, such as the transition of youth to adult healthcare systems [38], insulin management plans [39], or asthma management guidelines [42]. Multiple session programs covered a series of related topics or the same topic differently. The majority took the former approach, such as Caton et al.’s study on inpatient clinical scenarios and related team communication [30] or Ardoin et al.’s study on four common internal medicine cases for their new PGY-1 residents [27]. Similarly, Vertrees et al. described a series of PBL sessions on medical ethics, each with a theme taught through a pair of related cases [40]. The second approach was to have between two and four sessions on the same topic before changing to another one [32,33].

Of the studies that did describe their course development, two were primarily driven by residents [26,32] and one by a team that included upper-year subspecialty fellows, attending physicians, and the program director [39]. Some programs had content based specifically on needs assessments of their program’s PGME learners and supervisors [29,41]. Other studies described their PBL cases as tailored to the local context and patient population [28].

Learners were actively involved in the development of PBL, particularly through case selection [28,36,40]. This learner-driven approach was confirmed by feedback from the courses, which indicated a strong interest in real, resident-generated cases as a foundation for PBL [29]. This approach not only enhances learner engagement but also ensures that the cases are relevant to their community or medical service, thereby enriching the learning experience.

Training Program Facilitator 

PGME facilitators for these PBL sessions can be divided into experts or non-experts. Four sessions were led by subject experts, such as a behavioral and mental health assessment session for pediatric residents led by a child and adolescent psychiatry fellow [35]. Similarly, Schulmeister et al. and El-Hallal et al. had sessions for pediatric residents led by a pediatric epilepsy fellow [33,39]. In contrast, some sessions were led by non-experts to encourage peer learning [42]. Other sessions had facilitators senior to the learners but not experts, such as senior residents for PGY-1 interventions [27,30].

Level 1 Outcome: Learner Satisfaction 

Learners overwhelmingly expressed satisfaction with the PBL training programs, finding them relevant and engaging [27,29,38-40,42]. For instance, Caton et al. reported that 92% of respondents recommended the program’s continuation [30]. Similarly, three-quarters of the residents surveyed by Manning et al. rated the training in the top 5-10% of training experiences [35]. While some studies reported average feedback, with some learners showing less interest in further PBL opportunities [28,41], the overall high level of satisfaction underscores the effectiveness and relevance of PBL programs.

Level 2 Outcome: Change in Opinion, Knowledge, and/or Skill

Change in opinion, knowledge, and skill was measured in two ways: subjectively and objectively. Subjectively, learners found PBL more efficacious and engaging than other modalities [27,28,41]. Studies also reported increased understanding following the PBL session [28,30]. For example, Ramadurai et al. showed that 85% of learners could identify the impact of social determinants of health on care which increased to 100% of learners, post-intervention [36]. Respondents also indicated increased self-efficacy and confidence, as reported in four of five studies examining this metric [32,37,39,42]. Manning et al. found that the greatest confidence gains were in subjects the learners were already moderately comfortable with, rather than those already well-known or extremely difficult [35]. More broadly, Sass et al. reported that learners gain a deeper appreciation for social and economic factors related to health [37]. In summary, subjective measurements of opinion, knowledge, and skill changes demonstrate largely positive outcomes. 

Several studies conducted objective measurements showing increased knowledge among learners who completed the intervention [29,36,39]. Studies suggest that a certain baseline knowledge level predisposes learners to show significant knowledge gains, such as Harris et al., which reported significant gains for PGY-1 residents but not upper years [34].

Level 3 Outcome: Behaviour Change

While none of the studies measured behavior change objectively, over half reported increased comfort in diagnosing, prescribing, and managing patients [29,33,38]. Some studies noted learners’ anticipated behavior changes, such as following updated practice guidelines [42] or managing suicide risk assessments [35].

Discussion

Compared to the existing literature, this review supports the positive feedback found from PBL in UGME settings, including subjective learner satisfaction, perceived knowledge gain, and objective learning outcomes. These findings are extended to PGME learning, highlighting the suitability of PBL for primary care due to the increased confidence it provides learners in handling important topics. For example, residents reported being more likely to manage behavioral and mental health conditions themselves after PBL sessions, rather than referring to specialists [35]. This suggests that PBL empowers primary care providers to practice to a fuller scope.

These studies revealed diversity in session structure, suggesting that tailoring the schedule to the topic and group needs is reasonable. Repetition of the topic or theme over several sessions proved effective, with learners becoming more comfortable in subsequent sessions [40], though this may reduce the breadth of topics addressed throughout the course of the program. Aligning PBL with specific rotations was also well-received, as it demonstrated the clear relevance of the content to practice [36].

Learner participation in session development emerged as a common feature. Learners often submitted cases for discussion, ensuring the relevance of the topic. If not possible, cases should at least reflect learner needs. Several of the interventions were facilitated by more senior residents, which both reinforced content for the learners and provided teaching experience for the senior residents [30], suggesting dual benefits to near-peer-led groups. Iterative feedback and adaptation are crucial to optimizing PBL for specific institutions, programs, and cohorts.

Strengths and Limitations 

A strength of this review is the variety of specialties, program years, topics, and session formats represented. This breadth allows for a comprehensive understanding of PBL in PGME. Another key strength is the inclusion of evaluation components across studies, allowing assessment of the interventions’ impact.

However, this review had a few important limitations. One limitation was the lack of control groups, making distinguishing benefits from PBL versus other learning opportunities difficult. Additionally, most evaluations were subjective, lacking objective, validated data on knowledge gained. The heterogeneity of interventions also complicates drawing definitive conclusions about specific PBL recommendations. Further, none of the included articles featured virtual-only interventions, which limits the generalizability of these findings from in-person programming to virtual settings.

Finally, most evaluations focused on learner satisfaction (Kirkpatrick Level 1) and changes in opinion, knowledge, or skills (Level 2). There was little evaluation of behavior change (Level 3) and none of patient outcomes (Level 4). Therefore, while earlier levels are more confidently reported, the actual impact of PBL on tangible workplace outcomes remains to be seen.

## Conclusions

This rapid review supports the use of PBL in PGME settings, highlighting its effectiveness and high learner satisfaction. Studies that included objective measurements reported an association between PBL and increased knowledge scores. Tailoring session formats and schedules to the specific needs of the learners may enhance the effectiveness of PBL. Repeating topics can reinforce learning, though it may limit the breadth of topics addressed. Aligning PBL with specific rotations and involving learners in case selection were particularly successful strategies, ensuring engagement and relevance of the sessions. Near-peer facilitation not only reinforced learning but also provided teaching experience for senior residents, suggesting dual benefits to this approach. Iterative feedback and adaptation are crucial to optimizing PBL for individual institutions, programs, and cohorts. These findings underscore the suitability of PBL for primary care, primarily due to the increased confidence of learners.

Future research should focus on conducting more rigorous studies with control groups to explore the specific impact of PBL versus other instructional methods, particularly in terms of behavior change and patient outcomes.

## Appendices

Appendix 1: Search strategy

Database: PubMed

Date: July 4, 2024

Search string: (("Education, Medical, Graduate"[Mesh] OR "post-graduate medical education"[tiab] OR "postgraduate medical education"[tiab] OR PGME[tiab] OR "medical residen*"[tiab] OR "medical intern*"[tiab] OR "graduate medical education"[tiab] OR "fellowship*"[tiab]) AND ("Problem-Based Learning"[Mesh] OR "PBL"[tiab] OR "case-based learning"[tiab] OR "CBCL"[tiab] OR "case based collaborative learning"[tiab] OR "problem based learning"[tiab] OR "problem based"[tiab])) AND (English[la])

## References

[1] Barrows HS, Neufeld VR (1974). The “McMaster philosophy”: an approach to medical education. J Med Educ.

[2] Baum KD, Axtell S (2005). Trends in North American medical education. Keio J Med.

[3] Karimi R (2011). Interface between problem-based learning and a learner-centered paradigm. Adv Med Educ Pract.

[4] Riddell J, Jhun P, Fung CC (2017). Does the flipped classroom improve learning in graduate medical education?. J Grad Med Educ.

[5] Boshoff K, Murray C, Worley A, Berndt A (2020). Interprofessional education placements in allied health: a scoping review. Scand J Occup Ther.

[6] Joshi T, Budhathoki P, Adhikari A, Poudel A, Raut S, Shrestha DB (2021). Improving medical education: a narrative review. Cureus.

[7] Preeti B, Ashish A, Shriram G (2013). Problem based learning (PBL) - an effective approach to improve learning outcomes in medical teaching. J Clin Diagn Res.

[8] Dochy F, Segers M, Van den Bossche P, Gijbels D (2003). Effects of problem-based learning: a meta-analysis. Learn Instr.

[9] Yew EH, Goh K (2016). Problem-based learning: an overview of its process and impact on learning. Health Prof Educ.

[10] Albanese MA, Mitchell S (1993). Problem-based learning: a review of literature on its outcomes and implementation issues. Acad Med.

[11] Weggemans MM, van Dijk B, van Dooijeweert B, Veenendaal AG, Ten Cate O (2017). The postgraduate medical education pathway: an international comparison. GMS J Med Educ.

[12] Batalden MK, Warm EJ, Logio LS (2013). Beyond a curricular design of convenience: replacing the noon conference with an academic half day in three internal medicine residency programs. Acad Med.

[13] Eid A, Hsieh P, Shah P, Wolff R (2015). Cross-sectional longitudinal study of the academic half-day format in a hematology-oncology fellowship training program. BMC Med Educ.

[14] Ha D, Faulx M, Isada C (2014). Transitioning from a noon conference to an academic half-day curriculum model: effect on medical knowledge acquisition and learning satisfaction. J Grad Med Educ.

[15] Moreno MA, Kota R, McIntosh GC, Frohna JG (2013). PEARLs of wisdom: impact of a new block conference on pediatrics resident attendance, satisfaction, and learning. J Grad Med Educ.

[16] Cooper AZ, Hsieh G, Kiss JE, Huang GC (2017). Flipping out: does the flipped classroom learning model work for GME?. J Grad Med Educ.

[17] King AM, Gottlieb M, Mitzman J, Dulani T, Schulte SJ, Way DP (2019). Flipping the classroom in graduate medical education: a systematic review. J Grad Med Educ.

[18] King A, Boysen-Osborn M, Cooney R (2017). Curated collection for educators: five key papers about the flipped classroom methodology. Cureus.

[19] Wittich CM, Agrawal A, Wang AT (2018). Flipped classrooms in graduate medical education: a national survey of residency program directors. Acad Med.

[20] Newton WP (2011). Family physician scope of practice: what it is and why it matters. J Am Board Fam Med.

[21] Jewell K, Newton C, Dharamsi S (2015). Length of family medicine training and readiness for independent practice: residents’ perspectives at one Canadian university. UBCMJ.

[22] Kirkpatrick D (1996). Great ideas revisited: techniques for evaluating training programs. Train Dev.

[23] Steinert Y, Mann K, Centeno A, Dolmans D, Spencer J, Gelula M, Prideaux D (2006). A systematic review of faculty development initiatives designed to improve teaching effectiveness in medical education: BEME Guide No. 8. Med Teach.

[24] Jones PD, Seoane L, Deichmann R Jr, Kantrow C (2011). Differences and similarities in the practice of medicine between Australia and the United States of America: challenges and opportunities for the University of Queensland and the Ochsner Clinical School. Ochsner J.

[25] Hong QN, Pluye P, Fabregues S (2024). Mixed methods appraisal tool (MMAT) version 2018: user guide. Canadian Intellectual Property Office, Industry Canada.

[26] Agrawal S, Saluja I, Kaczorowski J (2004). A prospective before-and-after trial of an educational intervention about pharmaceutical marketing. Acad Med.

[27] Ardoin TW, Hamer D, Stumpf M, Miles L (2022). Integrating problem-based learning into an internal medicine residency curriculum. Ochsner J.

[28] Benson BL, Ha M, Stansfield RB, Markova T (2018). Health disparities educational initiative for residents. Ochsner J.

[29] Blair RA, Caton JB, Hamnvik OR (2020). A flipped classroom in graduate medical education. Clin Teach.

[30] Caton JB, Penn EH, Nemer MK, Katz JT, Yialamas MA (2019). Getting up to speed: a resident-led inpatient curriculum for new internal medicine interns. MedEdPORTAL.

[31] Dickerman J, Sánchez JP, Portela-Martinez M, Roldan E (2018). Leadership and academic medicine: preparing medical students and residents to be effective leaders for the 21st century. MedEdPORTAL.

[32] Dittus C, Grover V, Panagopoulos G, Jhaveri K (2014). Chief's seminar: turning interns into clinicians. F1000Res.

[33] El-Hallal M, Steiner S, Pavkovic I (2023). Project CARPE diem: curriculum and assessment for residents on pediatric epilepsy. Pediatr Neurol.

[34] Harris KI, Rowat JS, Suneja M (2020). Embedding a longitudinal diagnostic reasoning curriculum in a residency program using a bolus/booster approach. Diagnosis (Berl).

[35] Manning A, Weingard M, Fabricius J, French A, Sendak M, Davis N (2023). Be ExPeRT (behavioral health expansion in pediatric residency training): a case-based seminar. MedEdPORTAL.

[36] Ramadurai D, Sarcone EE, Kearns MT, Neumeier A (2021). A case-based critical care curriculum for internal medicine residents addressing social determinants of health. MedEdPORTAL.

[37] Sass P, Edelsack P (2001). Teaching community health assessment skills in a problem-based format. Acad Med.

[38] Sathe M, Werzen AS, Davis N, Millstein LS (2022). Implementing a longitudinal adolescent transition of care curriculum: identifying comfort and barriers among residents. Cureus.

[39] Schulmeister C, Laves E, Wong J, Walch A (2023). Pediatric resident insulin management education (PRIME): a single-session workshop emphasizing active learning. MedEdPORTAL.

[40] Vertrees SM, Shuman AG, Fins JJ (2013). Learning by doing: effectively incorporating ethics education into residency training. J Gen Intern Med.

[41] Volerman A, Poeppelman RS (2019). A pilot study of team-based learning in one-hour pediatrics residency conferences. BMC Med Educ.

[42] Warman KL, Silver EJ (2023). Get SMART: teaching pediatric residents the 2020 focused asthma updates’ recommendations for symptom-based medication increases. MedEdPORTAL.

